# A novel nomogram containing efficacy indicators to predict axillary pathologic complete response after neoadjuvant systemic therapy in breast cancer

**DOI:** 10.3389/fendo.2022.1042394

**Published:** 2022-11-25

**Authors:** Wenjie Shi, Xiaofeng Huang, Ye Wang, Xinyu Wan, Jinzhi He, Yinggang Xu, Weiwei Zhang, Rui Chen, Lu Xu, Xiaoming Zha, Jue Wang

**Affiliations:** ^1^ Department of Breast Disease, The First Affiliated Hospital of Nanjing Medical University, Nanjing, China; ^2^ Department of Clinical Nutrition, The First Affiliated Hospital of Nanjing Medical University, Nanjing, China

**Keywords:** Breast cancer, neoadjuvant systemic therapy, efficacy indicators, pathologic complete response, nomogram

## Abstract

**Background:**

Neoadjuvant systemic therapy (NST) could make some clinically node-positive (cN+) breast cancer patients achieve axillary pathologic complete response (pCR). This study aimed to identify the patients who are likely to achieve axillary pCR and help surgeons make surgical decisions on the axilla.

**Methods:**

The cN+ breast cancer patients who received NST from 2015 to 2021 at The First Affiliated Hospital of Nanjing Medical University were enrolled. Univariate and multivariate logistic regression analyses were performed, and a nomogram was constructed based on the results of multivariate logistic regression analysis to predict the probability of axillary pCR and validated.

**Results:**

The axillary pCR was achieved in 208 (38.7%) patients. Patients who had a higher radiological response rate of breast tumor (*P* = 0.039), smaller longest diameter of positive node after NST (*P* = 0.028), ER-negative status (*P* = 0.006), HER2-positive status (*P* = 0.048) and breast pCR (*P* < 0.001) were more likely to achieve axillary pCR. The nomogram had an area under the receiver operating characteristic curve (AUC) of 0.792 (95% CI: 0.744–0.839), and the calibration curve showed good agreement.

**Conclusion:**

A nomogram was constructed to predict the axillary pCR of cN+ patients receiving NST based on baseline and efficacy indicators to assist surgeons in making surgical decisions on the axilla.

## Introduction

Currently, breast cancer is one of the most common malignant tumors and one of the leading causes of death in women worldwide (1). Surgeons are increasingly embracing the idea of neoadjuvant systemic therapy (NST), because for inoperable disease, NST can decrease tumor burden and increase the eligibility of patients for surgery. In addition, NST may also provide breast-conservation opportunities and de-escalate axillary surgery for patients who were previously unable to receive such treatment (2–4).

Axillary lymph node metastasis is one of the most important prognostic factors for breast cancer. For patients with axillary lymph node metastasis, the recurrence rate is higher, and the survival time is shorter (5, 6). After NST, sentinel lymph node biopsy (SLNB) can be generally performed for initially clinically node-negative (cN-) patients, whereas for initially clinically node-positive (cN+) patients, axillary lymph node dissection (ALND) remains the standard surgical method (7). ALND is associated with many physical and psychological obstacles to patients, including upper extremity edema, numbness, stiffness, movement disorders, and psychological trauma (8–10). As a result, many efforts have been made to de-escalate axillary surgery after NST. The ACOSOG Z1071 trial concluded that SLNB may also be applied to cN+ breast cancer patients if removing the clipped node located before NST, simultaneously combining blue dye with radiolabeled colloid and examining at least three sentinel lymph nodes (11, 12). However, due to the higher price of the clips and the difficulty of obtaining radiolabeled colloids, the development and application of this technology in many countries is limited.

In order to find a convenient, accurate, and more economical method to predict axillary pathologic complete response (pCR), many predictive models have been developed (13–16). It should be noted that, these models were based more on the baseline indicators before NST. However, different individuals have different responses to NST even if they have similar baseline indicators. The advantage of NST is that the efficacy indicators can be monitored, such as size of the primary tumor and the axillary lymph nodes. These efficacy indicators may also be important for evaluating response to NST and predicting axillary pCR.

In this study, cN+ patients who had previously received NST for breast cancer in our center were enrolled. Baseline indicators before NST and efficacy indicators after NST of patients were all collected and analyzed. Patients were randomly divided into a training set and a validation set. Then, a nomogram to predict the axillary pCR was established based on both baseline and efficacy indicators in the training set, which were then confirmed in the validation set. This nomogram may be used to help surgeons make surgical decisions regarding axilla.

## Materials and methods

### Patient population

This retrospective study was approved by the Ethics Review Committee (2022-SR-201). We collected data from breast cancer patients who previously received NST at The First Affiliated Hospital of Nanjing Medical University from 2015 to 2021. The eligibility criteria for this study were as follows (1): diagnosed with primary unilateral breast cancer (2), received NST (3), tumor size was measurable (4), all patients were cN+ (5), all patients underwent mastectomy or lumpectomy combined with ALND, and (6) postoperative pathology information for breast and axilla was complete. Patients with missing data were excluded.

A total of 676 breast cancer patients receiving NST from 2015 to 2021 at The First Affiliated Hospital of Nanjing Medical University were enrolled. Of these, 538 patients were eligible to enter our study. We used a computer to assign a random number to each patient, scrambled the number, and divided it into a training set and a validation set at a ratio of 7:3. Finally, 378 and 160 patients were respectively enrolled in the training and validation set.

### Clinicopathologic evaluation

The maximum diameter of breast tumor and the longest diameter of positive node were measured by ultrasound or MRI before and after NST. Whether ultrasound or MRI was used, the same examination was used to evaluate response both before and after NST. The radiological response rate of breast tumor was considered the reduction rate of the maximum diameter of the tumor after NST compared with the maximum diameter of the tumor before NST. The radiological response rate of positive node was the reduction rate of the longest diameter of positive node after NST compared with the longest diameter of positive node before NST. The response evaluation of breast tumor was measured according to RECIST (version 1.1) (17). The clinical and pathologic stage of primary tumor was assessed according to the eighth edition of the AJCC cancer staging system (18).

Before NST, we performed a needle biopsy of the tumor for immunohistochemical analysis. We considered ER and PgR assays to be positive if there were at least 1% positive nuclear-stained cells in the tumor (19). The tumors with an immunohistochemistry [IHC] score 3+ or IHC2+/*in situ* hybridization [ISH]-positive were designated HER2 positive (20). Ki67 expression > 20% was considered as a high Ki67 level (21). The patients primarily received three NST regimens, which included epirubicin and cyclophosphamide followed by taxanes (EC-T), epirubicin and cyclophosphamide followed taxanes and trastuzumab (EC-TH), and epirubicin and cyclophosphamide followed by taxanes, trastuzumab and pertuzumab (EC-THP). Only trastuzumab was administered to some HER2-positive patients in the present study, because pertuzumab entered the China Medical Insurance List for the first time on 1 January 2020. Breast pCR (ypT0/is) was defined as the absence of residual invasive carcinoma in the breast on postoperative pathology after NST, irrespective of *in situ* ductal carcinoma (22).

### Statistical analysis

For the general characteristics of the patients, continuous variables are presented as means and standard deviations, and categorical variables are presented as frequencies and proportions. At first, the univariate logistic regression analysis was performed to screen out the variables associated with axillary pCR after NST. Then, variables with *P* < 0.05 were included in the multivariate logistic regression analysis. Odds ratios (ORs) and 95% CIs were also calculated.

The final nomogram was developed by the variables that were statistically significant. After the model was established, the predictive ability of the model was determined through discrimination and calibration. The discrimination ability of the model was assessed by the receiver operating characteristic (ROC) curve area under the curve (AUC), which ranged from 0.5 to 1.0. The 0.5 value indicates a random chance and 1.0 indicates a perfect fit. The calibration ability was primarily evaluated using a calibration curve. All statistical analyses were performed using IBM SPSS Statistics 21.0 software (IBM Corporation, Armonk, NY, USA), GraphPad Prism version 9.0.0 (GraphPad Software, San Diego, California USA), and R version 4.0.5 software (The R Foundation for Statistical Computing, Austria, Vienna).

## Results

### Patient characteristics

**Figure 1:**
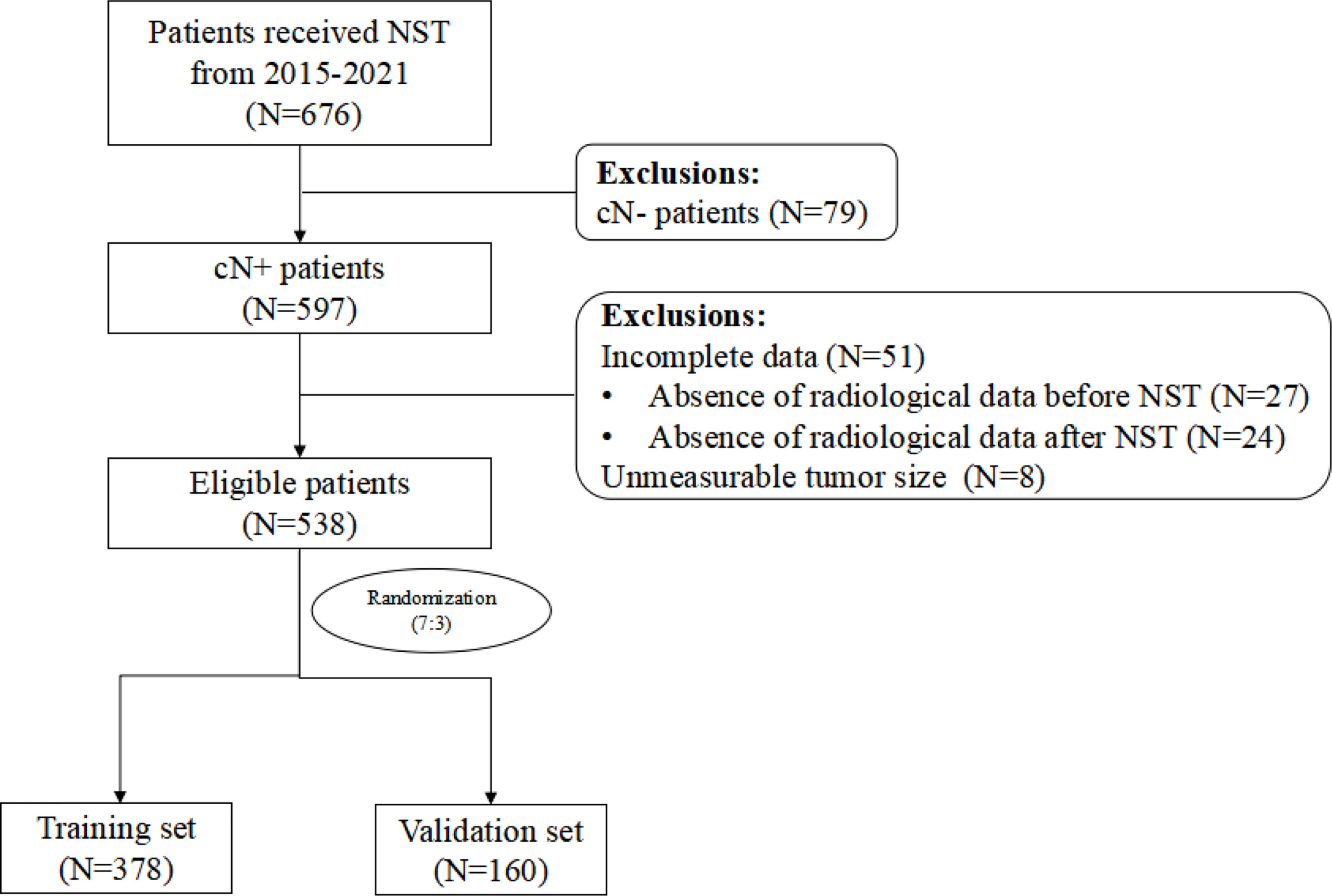
Study flowchart NST, neoadjuvant systemic therapy; cN-, clinically node-negative; cN+, clinically node-positive.

The study flowchart is shown in **Figure 1**. The clinicopathologic characteristics before and after NST for the patients in the training set, validation set, and total population are listed in **Table 1**.

**Table 1:** Characteristics of patients in the training set, validation set and total population.

Characteristics	Training set N = 378 (%)	Validation setN = 160 (%)	TotalN = 538 (%)	*P-*value
Age at diagnosis (years)	49.2 ± 10.9	49.0 ± 9.2	49.1 ± 10.4	0.430
Menstrual status				0.629
Premenopausal	186 (49.2)	86 (53.8)	272 (50.6)	
Postmenopausal	192 (50.8)	74 (46.2)	266 (49.4)	
Location of tumor				0.146
Left breast	198 (52.4)	69 (43.1)	267 (49.6)	
Right breast	180 (47.6)	91 (56.9)	271 (50.4)	
Clinical tumor size (cT)				0.872
cT1	31 (8.2)	16 (10.0)	47 (8.7)	
cT2	259 (68.5)	99 (61.9)	358 (66.5)	
cT3	51 (13.5)	28 (17.5)	79 (14.7)	
cT4	37 (9.8)	17 (10.6)	54 (10.0)	
Maximum diameter of breast tumor (mm)				
Before NST	39.3 ± 18.0	39.6 ± 18.1	39.4 ± 18.0	0.983
After NST	19.2 ± 15.3	20.0 ± 16.9	19.4 ± 15.8	0.853
Radiological response rate of breast tumor (%)	51.4 ± 30.3	49.3 ± 33.3	50.7 ± 31.2	0.774
Response evaluation of breast tumor				0.673
Complete response (CR)	46 (12.2)	15 (9.4)	61 (11.3)	
Partial response (PR)	250 (66.1)	103 (64.4)	353 (65.6)	
Stable disease (SD)	78 (20.6)	37 (23.1)	115 (21.4)	
Progressive disease (PD)	4 (1.1)	5 (3.1)	9 (1.7)	
Longest diameter of positive node (mm)				
Before NST	19.2 ± 9.4	19.1 ± 9.4	19.2 ± 9.4	0.998
After NST	11.4 ± 6.6	11.9 ± 7.4	11.6 ± 6.8	0.753
Radiological response rate of positive node (%)	36.4 ± 29.6	33.9 ± 39.3	35.6 ± 32.7	0.708
ER status				0.716
Negative	144 (38.1)	55 (34.4)	199 (37.0)	
Positive	234 (61.9)	105 (65.6)	339 (63.0)	
PgR status				0.295
Negative	205 (54.2)	75 (46.9)	280 (52.0)	
Positive	173 (45.8)	85 (53.1)	258 (48.0)	
HER2 status				0.980
Negative	235 (62.2)	98 (61.2)	333 (61.9)	
Positive	143 (37.8)	62 (38.8)	205 (38.1)	
Ki67 expression				0.618
≤ 20%	45 (11.9)	24 (15.0)	69 (12.8)	
> 20%	333 (88.1)	136 (85.0)	469 (87.2)	
NST regimens				0.718
EC-T	223 (59.0)	95 (59.4)	318 (59.1)	
EC-TH(P)	129 (34.1)	59 (36.9)	188 (34.9)	
Others	26 (6.9)	6 (3.8)	32 (5.9)	
Surgeries of breast				0.924
Mastectomy	351 (92.9)	147 (91.9)	498 (92.6)	
Lumpectomy	27 (7.1)	13 (8.1)	40 (7.4)	
Breast pCR (ypT0/is)				0.976
No	292 (77.2)	125 (78.1)	417 (77.5)	
Yes	86 (22.8)	35 (21.9)	121 (22.5)	

NST, neoadjuvant systemic therapy; ER, estrogen receptor; PgR, progesterone receptor; HER2, human epidermal growth factor receptor 2; EC-TH(P), epirubicin and cyclophosphamide followed by taxane, trastuzumab and pertuzumab; pCR, pathologic complete response; ypT0/is, the absence of residual invasive carcinoma in the breast on postoperative pathology after NST, irrespective of in situ ductal carcinoma.

The mean age of the 538 patients was 49.1 years. Premenopausal and postmenopausal patients accounted for approximately 50%. The proportion of left and right breast cancers were essentially equal (49.6% vs. 50.4%). There were the largest number of patients with cT1 and cT2 (75.2%). The mean maximum diameter of breast tumor was 39.4 mm before NST and 19.4 mm after NST. The mean radiological response rate of breast tumor was 50.7%. According to the response evaluation of breast tumor, only nine (1.7%) patients had progressive disease (PD) after NST. The mean longest diameter of positive node was 19.2 mm before NST and 11.6 mm after NST. The mean radiological response rate of positive node was 35.6%. ER status was positive in 339 (63.0%) patients, and PgR status was positive in 258 (48.0%) patients. HER2 status was positive in 205 (38.1%) patients. The vast majority of patients had high Ki67 expression (87.2%). With respect to the choice of NST, most HER2-negative patients received EC-T as the NST regimen. Of the 205 HER2-positive patients, more than 188 patients received targeted therapy during NST. There were also a small number of HER2-positive patients who refused targeted therapy. Regarding the choice of surgery, 498 (92.6%) patients selected mastectomy, while only 40 (7.4%) patients chose lumpectomy. Finally, 121 (22.5%) patients achieved breast pCR.

### Clinicopathologic response to NST

**Figure 2:**
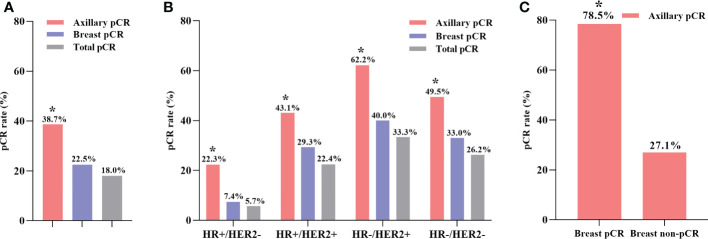
The axillary pCR rate was significantly higher than breast pCR rate in all patients **(A)**. The axillary pCR rate was significantly higher than breast pCR rate in different molecular subtypes of breast cancer **(B)**. The axillary pCR rate was significantly higher in the breast pCR subgroup than in the breast non-pCR subgroup **(C)**. *P < 0.05; pCR, pathologic complete response; HR, hormone receptor; HER2, human epidermal growth factor receptor 2.

As shown in **Figure 2A**, 208 (38.7%) patients achieved axillary pCR, 121 (22.5%) patients achieved breast pCR, and 95 (18.0%) patients achieved total pCR among all 538 cN+ patients after NST. Different molecular subtypes of breast cancer had different responses to NST (**Figure 2B**). HR-/HER2+ breast cancer patients exhibited the highest axillary pCR rate (62.2%), while HR+/HER2- patients had the lowest axillary pCR rate (22.3%). Additionally, regardless of the molecular subtypes of breast cancer, the axillary pCR rate was significantly higher compared with that of the breast pCR after NST (**Supplementary Tables 1, 2**). A subgroup analysis was performed on the basis of different breast pathologies after surgery (**Figure 2C**). Among 121 (22.5%) patients achieving breast pCR, 95 (78.5%) patients had simultaneous axillary pCR, whereas in 417 (77.5%) patients with breast non-pCR, only 113 (27.1%) patients achieved axillary pCR (*P* < 0.001, **Supplementary Table 3**).

### Significant predictors for axillary pCR

**Figure 3:**
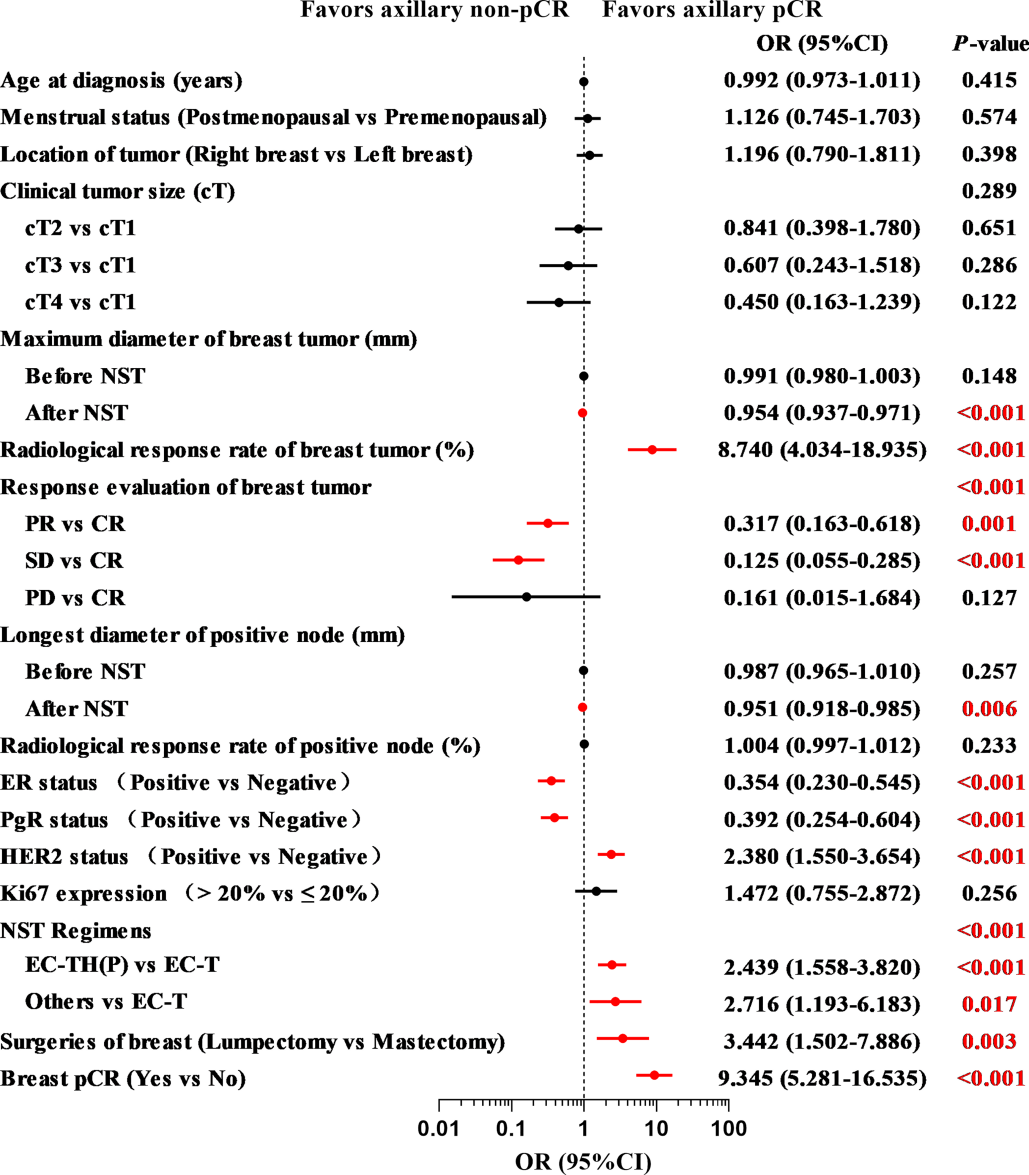
The results of univariate logistic regression analysis of indicators associated with axillary pCR in the training set (N = 378) pCR, pathologic complete response; NST, neoadjuvant systemic therapy; CR, complete response; PR, partial response; SD, stable disease; PD, progressive disease; ER, estrogen receptor; PgR, progesterone receptor; HER2, human epidermal growth factor receptor 2; EC-TH(P), epirubicin, cyclophosphamide followed by taxanes, trastuzumab and pertuzumab.

The results of univariate logistic regression analysis are shown in **Figure 3**. After NST, patients with a smaller maximum diameter of breast tumor (OR = 0.954, 95% CI: 0.937–0.971, *P* < 0.001), higher radiological response rate of breast tumor (OR = 8.740, 95% CI: 4.034–18.935, *P* < 0.001), and smaller longest diameter of positive node (OR = 0.951, 95% CI: 0.918–0.985, *P* = 0.006) were more likely to achieve axillary pCR. Compared with complete response (CR), patients with partial response (PR) or stable disease (SD) were less likely to achieve axillary pCR (OR = 0.317, 95% CI: 0.163–0.618, *P* = 0.001 and OR = 0.125, 95% CI: 0.055–0.285, *P* < 0.001). For patients with a negative ER status (OR = 0.354, 95% CI: 0.230–0.545, *P* < 0.001), a negative PgR status (OR = 0.392, 95% CI: 0.254–0.604, *P* < 0.001), and a positive HER2 status (OR = 2.380, 95% CI: 1.550–3.654, *P* < 0.001), the axillary pCR was more readily achieved. Patients who were treated with an EC-TH(P) regimen were more likely to achieve axillary pCR than EC-T regimen (OR = 2.439, 95% CI: 1.558–3.820, *P* < 0.001). Compared with mastectomy, patients choosing lumpectomy had a higher probability of achieving axillary pCR (OR = 3.442, 95% CI: 1.502–7.886, *P* = 0.003). According to postoperative pathology results, patients with breast pCR also had a higher probability to achieve axillary pCR than those with breast non-pCR (OR = 9.345, 95% CI: 5.281–16.535, *P* < 0.001). Multivariate logistic regression analysis was performed to identify variables that could predict axillary pCR after NST (**Table 2**). The variables related to axillary pCR were the radiological response rate of breast tumor (*P* = 0.039), the longest diameter of positive node after NST (*P* = 0.028), ER status (*P* = 0.006), HER2 status (*P* = 0.048) and breast pCR (*P* < 0.001).

**Table 2:** Multivariate logistic regression analysis of variables associated with axillary pCR in the training set (N=378).

Variable	OR (95%CI)	*P*-value
Radiological response rate of breast tumor (%)	2.531 (1.048 - 6.112)	0.039*
Longest diameter of positive node after NST (mm)	0.956 (0.918 - 0.995)	0.028*
ER status		0.006*
Negative	1.0	
Positive	0.502 (0.306 - 0.821)	
HER2 status		0.048*
Negative	1.0	
Positive	1.638 (1.004 - 2.673)	
Breast pCR		< 0.001*
No	1.0	
Yes	5.395 (2.896 - 10.049)	

pCR, pathologic complete response; NST, neoadjuvant systemic therapy; ER, estrogen receptor; HER2, human epidermal growth factor receptor 2; *Asterisks indicate statistically significant associations (p<0.05).

### Construction and validation of the nomogram

**Figure 4:**
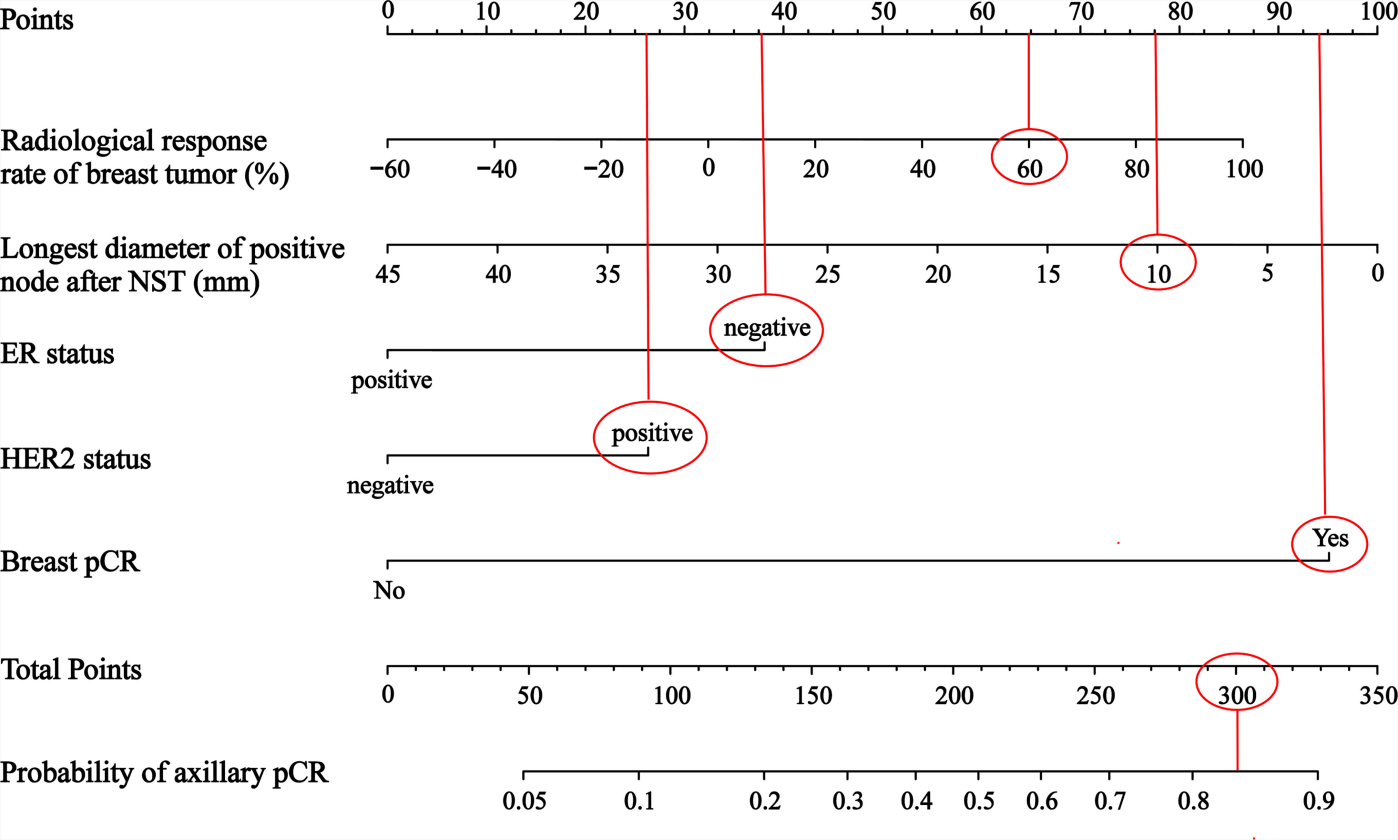
A nomogram to predict axillary pCR after NST in cN+ breast cancer patients NST, neoadjuvant systemic therapy; ER, estrogen receptor; HER2, human epidermal growth factor receptor 2; pCR, pathologic complete response.

A nomogram was constructed to predict the probability of achieving the axillary pCR after NST based on the results of multivariate logistic regression analysis, which included the baseline and efficacy indicators before and after NST (**Figure 4**). For example, for a patient, if the radiological response rate of breast tumor is 60%, the longest diameter of positive node after NST is 10mm, the ER status is negative, the HER2 status is positive, and the breast achieves pCR, the points are roughly 65, 77.5, 37.5, 26 and 94. The total points are 300, so the probability of axillary pCR is approximately 84%.

**Figure 5:**
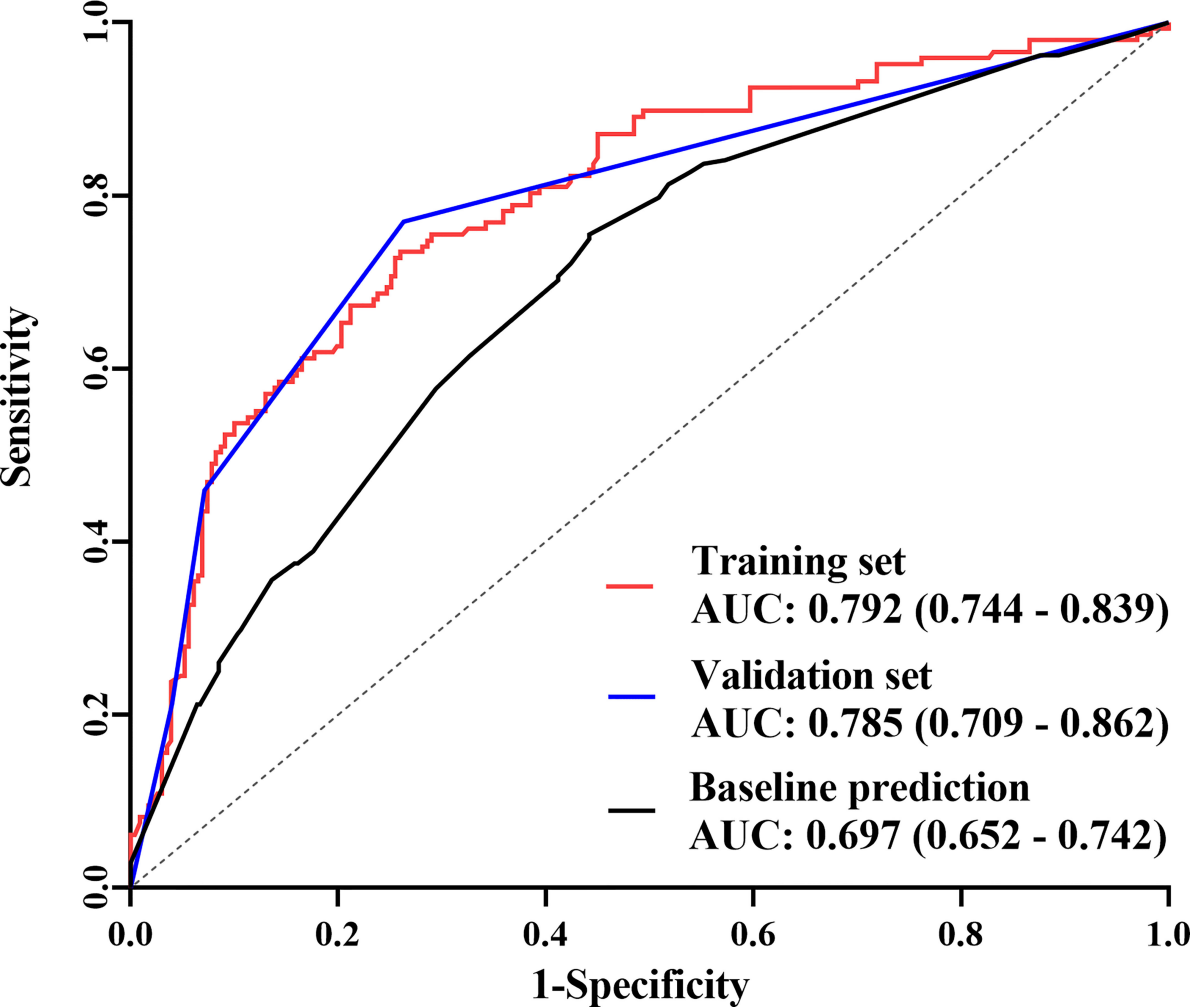
The receiver operating characteristic (ROC) curves The area under the curve (AUC) was 0.792 (95% CI: 0.744–0.839) in the training set, 0.785 (95% CI: 0.709–0.862) in the validation set, and 0.697 (95% CI: 0.652–0.742) in the predictive model only including baseline indicators.

**Figure 6:**
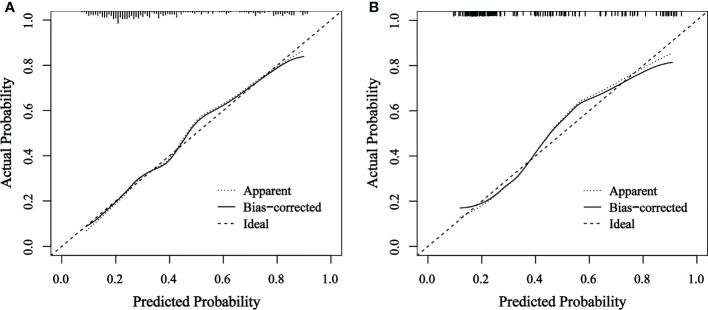
Calibration curve of actual and predicted probabilities of axillary pCR in **(A)** the training set and **(B)** the validation set The horizontal axis indicates the predicted probabilities measured by the nomogram and the vertical axis indicates the actual probabilities. pCR, pathologic complete response.

The final nomogram was verified using a bootstrap method (n=1000). The AUC of this predictive model was 0.792 (95% CI: 0.744–0.839) in the training set and 0.785 (95% CI: 0.709–0.862) in the validation set, which indicates that the model has stronger predictive power (**Figure 5**). And there was no statistical difference between these two AUCs (*P* = 0.882, Delong’s test) (23). The calibration curve used to assess the goodness-of-fit of the nomogram exhibited good agreement between the predicted and actual probabilities of axillary pCR in the training set and the validation set (**Figure 6**).

## Discussion

In this study, the axillary pCR rate (38.7%) was significantly higher than the breast pCR rate (22.5%) in all patients, which is consistent with other studies (24–26). These results indicate that axillary pCR is more common after NST. Thus, cN+ patients may be candidates for axillary de-escalation surgery. In a subgroup analysis, the axillary pCR rate of HR-/HER2+ breast cancer patients was the highest (62.2%) among the different molecular subtypes of breast cancer. In contrast, the axillary pCR rate of HR+/HER2- patients was the lowest (22.6%), which was similar to that of another report (60% for HR-/HER2+ and 18% for HR+/HER2-) (27). However, it is still worth noting that since the HR+/HER2- subtype accounts for the highest proportion (approximately 70%) of breast cancer (28), the total number of patients who finally achieved axillary pCR after NST is not small. As a result, a predictive model for axillary pCR is necessary for all molecular subtypes of breast cancer treated with NST.

To study the role of different indicators in predicting axillary pCR, enrolled patients were randomly assigned to training and validation sets at a ratio of 7:3. Based on the results of the multivariate logistic regression analysis, a final predictive model was constructed. Results of the univariate logistic regression analysis showed that most baseline indicators were not associated with axillary pCR, including age at diagnosis, menstrual status, and clinical tumor size. Efficacy indicators and some other baseline indicators were related to axillary pCR, so the multivariate logistic regression analysis was further conducted. The results indicated that some baseline indicators, including ER status and HER2 status, were associated with axillary pCR, which is consistent with other reports (13, 24, 29, 30). Different from previous findings, several efficacy indicators were found to be strongly associated with the axillary pCR, including the radiological response rate of breast tumor, the longest diameter of positive node after NST, and breast pCR. Results showed that the higher the radiological response rate of breast tumor after NST, the higher likelihood of achieving axillary pCR. Moreover, the smaller the longest diameter of positive node after NST, the higher probability of achieving axillary pCR. These two indicators were efficacy indicators that are closely associated with axillary pCR, which once again shows that the prediction of axillary pCR may focus not only on baseline indicators, but also on the efficacy indicators after NST.

In this study, the radiological response rate of breast tumor was associated with axillary pCR, so it was reasonable that breast pCR could predict axillary pCR as an extremity of the radiological response rate. Since this was a retrospective study, all enrolled patients had already received surgery and the pathological data for breast pCR had been obtained. However, this data is missing when this model is applied to the prediction of axillary pCR prior to surgery. At this time, if the breast clinical complete response (cCR) is not considered in preoperative radiological examinations, the probability of breast pCR is very small, therefore, it may be directly judged as breast non-pCR. On the other hand, if the breast cCR is highly probable based on preoperative radiological examinations, the pathological information can also be obtained before surgery. In a study aiming to omit breast surgery, when using image-guided vacuum-assisted biopsy to obtain at least six representative samples in patients with residual radiological abnormality or a tumor bed measuring 2 cm or smaller, the breast pCR can be accurately demonstrated (the false-negative rate was 3.2%) (31). In addition, intraoperative frozen pathology of breast tumors may also be applied during breast surgery, which provides the data for breast pCR to predict axillary pCR.

In contrast, the radiological response rate of positive node showed no correlation with axillary pCR. Unlike breast tumors, lymph nodes are inherently anatomical in size. The breast could achieve pCR when NST was highly effective, but the lymph nodes could only shrink back to their original size and would not disappear; thus, the radiological response rate of positive node could not achieve 100%. In this study, the mean longest diameter of positive node after NST was 11.6 mm, and the highest radiological response rate of positive node was 90%. This also suggests that evaluating the response of lymph nodes to NST may require the combination of other factors, such as the cortical thickness of the lymph nodes. As for the response evaluation of breast tumor, results showed that compared with PR and SD, patients achieving CR were more likely to achieve axillary pCR. While compared with PD, the predictive superiority of achieving CR was not demonstrated in this study. Since NST was effective, the number of PD patients was particularly small (1.7%), which affected the statistical results.

In this study, a nomogram containing efficacy indicators after NST was constructed. The AUC of this predictive model was 0.795 and the calibration curve also showed good agreement between the predicted and actual probabilities of axillary pCR, which indicated that the model had a more accurate prediction ability of axillary pCR. In contrast, when only baseline indicators were included, the AUC that was shown in **Figure 5** was 0.697 (*P* = 0.004, Delong’s test) (23), which was consistent with other report containing only baseline indicators (15). The above evidence confirmed that the efficacy indicators are crucial for predicting axillary pCR.

Our study also had some limitations. First, it was a retrospective and single-center study, and the number of patients used to construct and validate the nomogram was relatively small. Therefore, a subgroup analysis was somewhat limited. Second, for reasons of price and technique, fine-needle aspiration biopsy of axillary lymph nodes is not currently performed in many hospitals, and all of the many studies that have been published to date have been designed based on cN+ rather than pathologically node-positive (pN+) patients. Since last year, we have been performing fine-needle aspiration of positive lymph nodes before NST to clarify the status of the nodes. When the sample size is sufficient, we will design relevant clinical studies to predict axillary pCR after NST in pN+ patients.

In conclusion, we constructed a nomogram to predict axillary pCR after NST in cN+ patients with breast cancer. According to the results of the statistical analyses, efficacy indicators reflecting the therapeutic response of NST were found to be very important in predicting axillary pCR. Based on these efficacy indicators and some baseline indicators, we established a novel nomogram, which was validated and considered to be highly accurate in predicting axillary pCR. This predictive model may help surgeons to de-escalate or even omit axillary surgery for patients with axillary lymph node downstaging after NST in the future.

## Data availability statement

The original contributions presented in the study are included in the article/**Supplementary Material**. Further inquiries can be directed to the corresponding authors.

## Ethics statement

The studies involving human participants were reviewed and approved by Ethics Review Board of the First Affiliated Hospital of Nanjing Medical University. Written informed consent for participation was not required for this study in accordance with the national legislation and the institutional requirements.

## Author contributions

XZ and JW conceptualized and designed the study. WS, YW, XW, JH and RC completed the acquisition, analysis, and interpretation of the data. XZ, JW, and LX obtained the study funding. WS, XH, YX, and WZ were responsible for the methodology. XZ and JW provided study supervision. WS drafted the original version of the manuscript. All authors critically revised drafts of the manuscript and approved the final version.

## Funding

This research was partially supported by the Chinese Society of Clinical Oncology Foundation (Y-sy2018-077, Y-JS2019-096, Y-HR2020MS-0421), Jiangsu Province Elderly Health Research Project (LKM2022033), and Young Scholars Fostering Fund of the First Affiliated Hospital of Nanjing Medical University (PY2021038).

## Conflict of interest

The authors declare that the research was conducted in the absence of any commercial or financial relationships that could be construed as a potential conflict of interest.

## Publisher’s note

All claims expressed in this article are solely those of the authors and do not necessarily represent those of their affiliated organizations, or those of the publisher, the editors and the reviewers. Any product that may be evaluated in this article, or claim that may be made by its manufacturer, is not guaranteed or endorsed by the publisher.
