# Corrigendum: A novel nomogram containing efficacy indicators to predict axillary pathologic complete response after neoadjuvant systemic therapy in breast cancer

**DOI:** 10.3389/fendo.2022.1109390

**Published:** 2022-12-19

**Authors:** Wenjie Shi, Xiaofeng Huang, Ye Wang, Xinyu Wan, Jinzhi He, Yinggang Xu, Weiwei Zhang, Rui Chen, Lu Xu, Xiaoming Zha, Jue Wang

**Affiliations:** ^1^ Department of Breast Disease, The First Affiliated Hospital of Nanjing Medical University, Nanjing, China; ^2^ Department of Clinical Nutrition, The First Affiliated Hospital of Nanjing Medical University, Nanjing, China

**Keywords:** breast cancer, neoadjuvant systemic therapy, efficacy indicators, pathologic complete response, nomogram

In the published article, there was an error in the legend for **Figure 5** as published. The legend was incorrectly recorded as: “The receiver operating characteristic (ROC) curves The area under the curve (AUC) was 0.795 (95% CI: 0.747–0.843) in the training set, 0.809 (95% CI: 0.738–0.880) in the validation set, and 0.705 (95% CI: 0.651–0.759) in the predictive model only including baseline indicators.”

The corrected legend appears below:

“The receiver operating characteristic (ROC) curves The area under the curve (AUC) was 0.792 (95% CI: 0.744–0.839) in the training set, 0.785 (95% CI: 0.709–0.862) in the validation set, and 0.697 (95% CI: 0.652–0.742) in the predictive model only including baseline indicators.”

**Figure 5:**
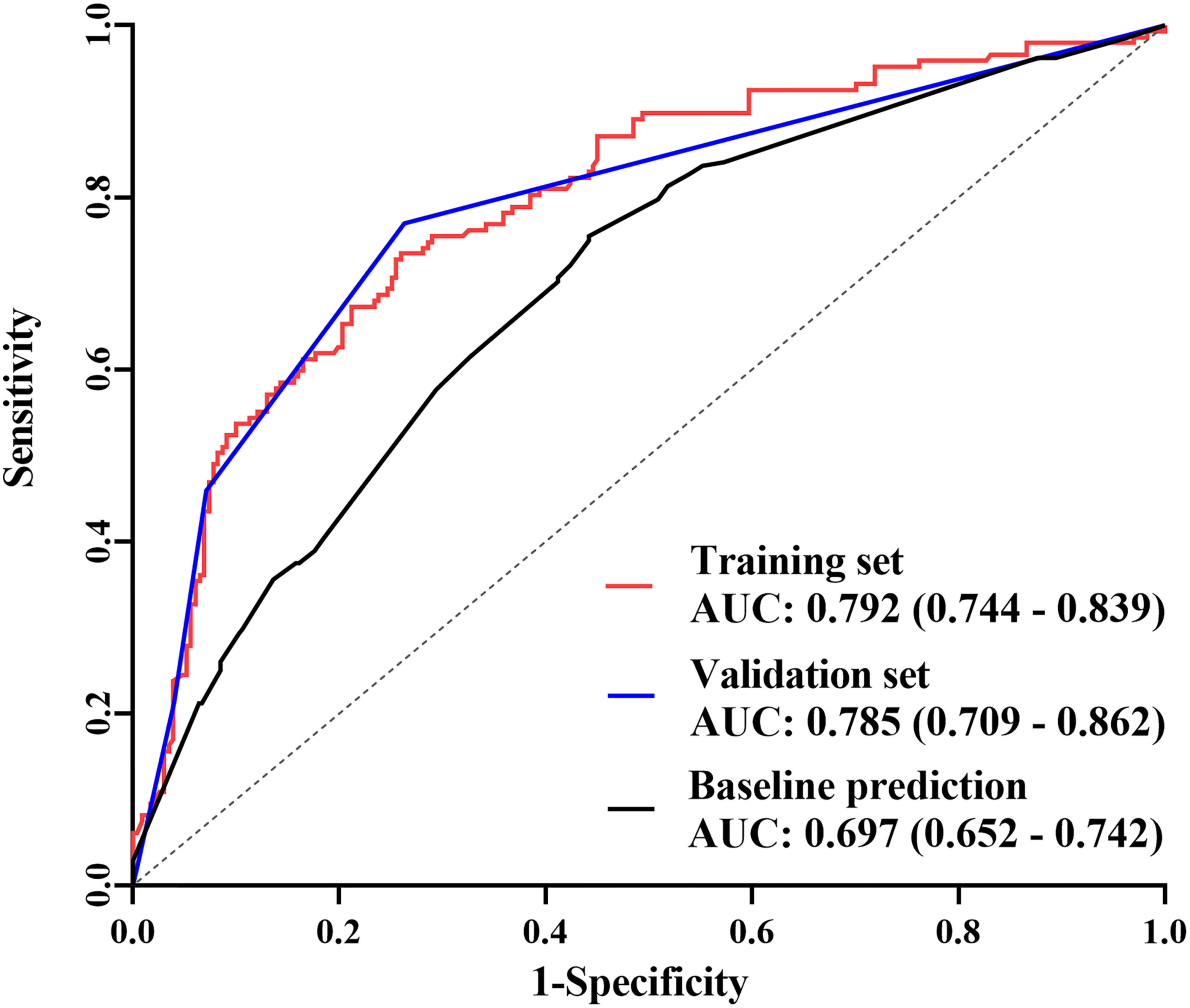
The receiver operating characteristic (ROC) curves The area under the curve (AUC) was 0.792 (95% CI: 0.744–0.839) in the training set, 0.785 (95% CI: 0.709–0.862) in the validation set, and 0.697 (95% CI: 0.652–0.742) in the predictive model only including baseline indicators.

In the published article, there was also an error in which the incorrect values were written. A correction has been made to **Abstract**, *Results*. This sentence previously stated:

“The nomogram had an area under the receiver operating characteristic curve (AUC) of 0.795 (95% CI: 0.747–0.843)”

The corrected sentence appears below:

“The nomogram had an area under the receiver operating characteristic curve (AUC) of 0.792 (95% CI: 0.744–0.839)”

The authors apologize for these errors and state they this does not change the scientific conclusions of the article in any way. The original article has been updated.

