# Supplementary material for: A novel nomogram containing efficacy indicators to predict axillary pathologic complete response after neoadjuvant systemic therapy in breast cancer
Source: Front Endocrinol (Lausanne). 2022 Nov 25;13:1042394. doi: 10.3389/fendo.2022.1042394 (PMC9732273; doi:10.3389/fendo.2022.1042394)
Supplement: Supplementary file 1 [file Table_1.doc]

***Supplementary materials***

**Supplementary Table 1.** The axillary pCR rate was significantly higher compared with the breast pCR rate in all patients.

| All patients | Yes (%) | No (%) | *P*-value |
| --- | --- | --- | --- |
| Axillary pCR | 208 (38.7) | 330 (61.3) | < 0.001 |
| Breast pCR | 121 (22.5) | 417 (77.5) |

**Abbreviations:** pCR, pathologic complete response

**Supplementary Table 2.** The axillary pCR rate was significantly higher compared with the breast pCR rate in different molecular subtypes of breast cancer.

| Molecular subtypes | | Yes (%) | No (%) | *P*-value |
| --- | --- | --- | --- | --- |
| HR+/HER2- | Axillary pCR | 51 (22.3) | 178 (77.7) | < 0.001 |
|  | Breast pCR | 17 (7.4) | 212 (92.6) |  |
| HR+/HER2+ | Axillary pCR | 50 (43.1) | 66 (56.9) | 0.029 |
|  | Breast pCR | 34 (29.3) | 82 (70.7) |  |
| HR-/HER2+ | Axillary pCR | 56 (62.2) | 34 (37.8) | 0.003 |
|  | Breast pCR | 36 (40.0) | 54 (60.0) |  |
| HR-/HER2- | Axillary pCR | 51 (49.5) | 52 (50.5) | 0.016 |
|  | Breast pCR | 34 (33.0) | 69 (67.0) |  |

**Abbreviations:** HR, hormone receptor; HER2, human epidermal growth factor receptor 2; pCR, pathologic complete response

**Supplementary Table 3.** The axillary pCR rate was significantly higher in the breast pCR subgroup than in the breast non-pCR subgroup.

| Breast pCR | Axillary pCR | | *P*-value |
| --- | --- | --- | --- |
|  | Yes (%) | No (%) |  |
| Yes | 95 (78.5) | 26 (21.5) | < 0.001 |
| No | 113 (27.1) | 304 (72.9) |  |

**Abbreviations:** pCR, pathologic complete response
